# Acceptability and Feasibility of the Plasma Separation Card for an Integrated Model of Care for HBV and HCV Screening Among People Attending HIV Clinics in Cameroon and Uganda

**DOI:** 10.1007/s44197-024-00220-w

**Published:** 2024-03-27

**Authors:** Camila A Picchio, Aina Nicolàs, Ironne Valdèse Ayemfouo Fofou, Viola Kasone, Magellan Guewo-Fokeng, Claude T Tagny, Teddy Nanyonjo, Hellen Nansumba, Yves Nacel Kouongni, Rita Gaëlle Epse Sezawo Kamdjeu, Emmanuel Seremba, Charles Kouanfack, Isaac Ssewanyana, Richard Njouom, Ariadna Rando Segura, Francisco Rodríguez-Frías, Jean Claude Mbanya, Ponsiano Ocama, Jeffrey V. Lazarus

**Affiliations:** 1 Barcelona Institute for Global Health (ISGlobal), Hospital Clínic, University of Barcelona, Barcelona, Spain; 2 Department of Biochemistry, Faculty of Science, University of Yaoundé I, Yaoundé, Cameroon; 3 Laboratory of Molecular Medicine and Metabolism, Biotechnology Centre, University of Yaoundé I, Yaoundé, Cameroon; 4 Central Public Health Laboratories, Ministry of Health of Uganda, Kampala, Uganda; 5 Hematology and Blood Transfusion Unit, University Teaching Hospital of Yaoundé, Yaoundé, Cameroon; 6 Kiruddu National Referral Hospital, Kampala, Uganda; 7 Day Hospital, HIV Care and Treatment Unit, Yaoundé Central Hospital, Yaoundé, Cameroon; 8 Department of Public Health, Faculty of Medicine and Pharmaceutical Sciences, University of Dschang, Dschang, Cameroon; 9 Centre Pasteur of Cameroon, Yaoundé, Cameroon; 10 Department of Microbiology, Vall d’Hebron Hospital Universitari, Barcelona, Spain; 11 CIBEREHD Instituto de Salud Carlos III, Madrid, Spain; 12 Liver Pathology Unit, Department of Biochemistry, Vall d’Hebron Institut de Recerca (VHIR), Barcelona, Spain; 13 Department of Internal Medicine and Specialties, Faculty of Medicine and Biomedical Sciences, University of Yaoundé I, Yaoundé, Cameroon; 14 Yaoundé Central Hospital, National Obesity Centre, University of Yaoundé I, Yaoundé, Cameroon; 15 College of Health Sciences, Makerere University, Kampala, Uganda; 16 City University of New York Graduate School of Public Health and Health Policy (CUNY SPH), New York, NY USA; 17 Faculty of Medicine, University of Barcelona, Barcelona, Spain

**Keywords:** Dried blood spot, Hepatitis B, Plasma separation card, Viral hepatitis testing, Cameroon, Uganda

## Abstract

**Background:**

Sub-Saharan African countries have a high burden of viral hepatitis and poor access to screening and care. The aim of this study was to evaluate the feasibility and acceptability of using the plasma separation card (PSC) for viral hepatitis B and C screening among people living with HIV (PLHIV) in Cameroon and Uganda.

**Methods:**

This is a cross-sectional study carried out between 05/2021 and 03/2023 including 192 PLHIV in Cameroon (*n* = 104) and Uganda (*n* = 88). Basic sociodemographic variables and whole blood samples were collected. Adequate filling with blood of PSCs was used to determine feasibility together with participant responses to questions on acceptability. A logistic regression model was carried out to assess the relationship between PSC acceptability and factors of interest.

**Results:**

70% of participants reported PSC as an acceptable viral hepatitis screening tool, and it was significantly more accepted in Uganda than Cameroon (100% vs. 43.2%, *p* < 0.001). Similarly, 75% of PSCs had at least one spot sample filled and were viable for analysis, 99% were correctly filled in Uganda and 53.4% in Cameroon. Reported ease of method performance (aOR: 24.77 95% CI 2.97-206.42, *p* = 0.003) and reduced collection time (aOR: 3.73 95% CI 1.26–11.04, *p* = 0.017) were associated with greater odds of PSC acceptance. HBsAg + and anti-HCV + prevalence were 11.1% and 1.0%, respectively.

**Conclusions:**

In spite of country differences, overall, the PSC was reported as a feasible and acceptable viral hepatitis testing method. Acceptability and feasibility of the method must be explored in heterogeneous target communities and qualitative research to better understand country-specific barriers and facilitators should be carried out.

## Introduction

Infections caused by the hepatitis B virus (HBV) and C virus (HCV) affect an estimated 290 million and 58 million people, respectively, around the world [1, 2]. In sub-Saharan Africa (sSA), HBV infection is endemic and the burden of HCV is also substantial [3]. The World Health Organization (WHO) estimates that 6% of the African population is HBsAg + and that HCV prevalence varies between 1% and 2% [2]. In Cameroon, the seroprevalence of HBV varies between regions, but a systematic review reports a pooled prevalence of 11.2% in the country [4]. Similarly, the overall pooled prevalence of anti-HCV antibodies has been found to be as high as 6.5% [4–6]. In Uganda, a systematic review describes a pooled HBV prevalence of 8.5% [7].

Persistent HBV or HCV infection can cause chronic inflammation of the liver, leading to fibrosis, cirrhosis and/or hepatocellular carcinoma. These infections caused 1.1 million yearly deaths between 2016 and 2021 [2], and are responsible for more than half of all liver cancer cases, the second biggest cancer cause of mortality in Africa [3]. Co-infection of viral hepatitis with HIV can accelerate significant liver fibrosis progression [8, 9]. Given its significant burden on health worldwide, the WHO set out to eliminate viral hepatitis as a public health threat by 2030 [10], by diagnosing 90% of viral hepatitis B and C cases.

To appropriately address viral hepatitis infections, adequate and timely diagnosis is needed. In low-resource settings, like in sSA, the lack of diagnostic infrastructure represents a major obstacle to viral hepatitis screening, therefore most HBV or HCV infections remain undiagnosed. Screening for HBV and HCV infection is carried out by assessing in serum or plasma for the presence of hepatitis B surface antigen (HBsAg) or anti-HCV antibodies (anti-HCV) followed by HBV-DNA and HCV-RNA detection if positive in the prior samples, respectively. However, virological tests are often inaccessible, limiting the access to any hepatitis services and thus denying proper disease management, including treatment, to a large proportion of patients. To overcome diagnostic challenges and facilitate HBV and HCV diagnoses in sSA, the dried blood spot (DBS) testing method can be employed. This method allows for greater facility in transportation and storage of samples and can be collected through fingerstick capillary whole blood [11, 12].

WHO guidelines encourage the use of DBS testing for HBV and HCV in regions that lack infrastructure or expertise for venous blood collection [13]. The performance of the DBS method has been previously validated in low and middle-income countries [14–17]. However, there is little evidence on its real-world implementation, feasibility, and patient acceptability. This is particularly the case for the plasma separation card (PSC), a DBS screening tool which further allows for the separation and stabilization of plasma from whole blood obtained through a blood draw or capillary blood via a filtration layer. The PSC currently has European (Conformité Européenne, CE) approval for HIV viral load and has been used in African settings for HIV viral load monitoring since 2017 [18, 19]. The PSC is a potential tool to help increase viral hepatitis testing and diagnoses in sSA. However, when reviewing the literature, only two of the more than 40 published reports of different viral hepatitis testing programs were from Africa [17], and only one [20] considered acceptability and feasibility of a rapid detection point-of-care test.

Studies involving the patient perspective and experience are lacking in sSA and there is a need to engage with patients when studying the feasibility of viral hepatitis testing strategies and methods. The objective of this study was to evaluate the feasibility and acceptability of using the PSC for viral hepatitis screening among PLHIV in Cameroon and Uganda.

## Methods

This is a cross-sectional study to evaluate the feasibility and acceptability of utilizing the PSC for viral hepatitis (HBV and HCV) testing in two sub-Saharan African countries which took place between May 2021 and March 2023. Testing in Kampala, Uganda, took place between 21 May and 4 June 2021 and testing in Yaoundé, Cameroon, was delayed until 14 March−23 March 2023 due to the COVID-19 pandemic, which restricted the ability for part of the study team to travel to provide training for the implementation of the study [21]. Patients attending HIV clinics for regular care in Kampala and Yaoundé were consecutively invited to participate in this study and were offered screening for HBV and HCV utilizing a sample (420µL) collected via capillary whole blood by trained nurses utilizing the plasma separation card (Roche Diagnostics, California) until the target number of 100 participants per site was reached. Inclusion criteria included being aged 18 or greater and receiving HIV care at one of the participating centres. Exclusion criteria included having a known viral hepatitis diagnosis and being too ill to participate. Selection of the study sites were based on previous and ongoing collaborations between the study team in addition to the availability of Roche Diagnostics platforms in the respective countries and facilities. All participants who tested positive in the study were informed of their status and referred to appropriate liver care specialists for follow-up.

### Procedure in Uganda

People attending Kiruddu National Referral Hospital (Kampala) for routine HIV care once per week (Fridays) were consecutively invited to participate in the study. After informed consent, samples were collected on the PSC from capillary whole blood and were allowed to dry for a minimum of 4 h, following the manufacturer’s protocol. Participants received a reimbursement for their transportation costs to attend the hospital. Samples were collected by trained laboratory staff, labelled with patient identifiers, dried for four hours using the drying rack, packed and transferred at the end of the day to the Central Public Health Laboratory (CPHL). Uganda has a centralized reference testing laboratory for viral hepatitis where all serology and molecular analyses were carried out. HBsAg and anti-HCV were determined with the COBAS®e411 platform (Roche Diagnostics) and all reactive samples were then analysed for HBV-DNA and HCV-RNA on the COBAS® 4800 (Roche Diagnostics).

### Procedure in Cameroon

People attending the Central Yaoundé I Hospital (Yaoundé) for routine HIV care every day (Monday−Friday) were consecutively invited to participate in the study. Samples were collected on the PSC from capillary whole blood and were allowed to dry for a minimum of 4 h, following the manufacturer’s protocol. Participants received a reimbursement for their transportation costs to attend the hospital. Samples were transported twice during the two-week period to the Hematology Laboratory of the University Teaching Hospital of Yaoundé (CHUY). Serology tests for HBsAg and anti-HCV were determined with the COBAS®e411 platform (Roche Diagnostics) at CHUY. Reactive samples were then shipped to the Liver Pathology Unit at Vall d’Hebron Hospital Universitari (Barcelona, Spain) for HBsAg confirmation and further molecular analysis (HBV-DNA and HCV-RNA) on the COBAS® 6800 (Roche Diagnostics) platform.

### Sociodemographic Information and Risk Factors

The sociodemographic variables collected were age, sex, ethnic group, number of people living in the same household, and level of education. Questions on herbal medicine use were collected in addition to possible risk factors for viral hepatitis infection, including recent hospitalization, prior surgery or blood transfusion, incarceration, and having tattoos or traditional scarring. Knowledge and history of previous testing of HBV and HCV were collected, in addition to self-reported HBV vaccination status (See Annex I).

### PSC Sample Extraction

Dried blood spot testing obtained from dried plasma samples with the PSC were collected by placing 140µL of fingerstick-collected whole blood onto special filter paper with three spots (420µL maximum volume) and were left to dry for a minimum of four hours. The PSC has an RNA stabilizer embedded in the card, reducing target degradation in the sample as compared to standard DBS papers. The PSC has demonstrated stability at 45ºC and 85% humidity combined for 28 days with additional desiccant added to the samples [18]. Once samples were received at the respective laboratories, viable blood spots were removed from the PSC using dressing forceps and processed following the established protocol for serological or molecular testing. For testing of HBsAg or anti-HCV (serology), the dried plasma samples were eluted and incubated at 37ºC with 600 µL of diluent (Elecsys universal diluent) overnight (at least 8 h) prior to being loaded onto the respective platforms (COBAS® e411) [22]. Following the HIV PSC method sheet, nucleic acids from the PSC were extracted for molecular testing and individual dried plasma spots were removed from the cards and incubated at 56ºC with 950 µL of specimen pre-extraction reagent (Roche®) for 10 min at 1000 rpm on a preheated thermoshaker and later loaded on the COBAS® 4800 and 6800 systems for nucleic acid extraction, amplification, and detection in accordance with the instructions for plasma using the 500 µL processing volume option.

### Acceptability and Feasibility

Participants were asked about the acceptability of this testing method, the level of pain experienced, the preferred sample collection duration, and their likelihood to recommend this sample collection method to others on a 5-point Likert scale. Acceptability was defined as the proportion of participants who ranked the method as “very acceptable,” “acceptable,” or “somewhat acceptable” on a 5-point Likert scale. Satisfaction was defined as the proportion of participants who reported being “very satisfied”, “satisfied”, or “somewhat satisfied” with the PSC method. Low level of pain was defined as “not at all painful”, “somewhat painful” or “neither painful or not painful” on a 5-point Likert scale in comparison to those who responded “somewhat painful”, and “very painful”. Feasibility was defined as the number of PSC samples for which at least one spot was correctly filled and an analysis could be performed. Laboratory personnel at each respective laboratory were responsible for determining and reporting the number of PSCs with adequately filled blood spots and with the required volume (*n* = 3 spots and 140 µL each). Duration of time (minutes) it took to collect the blood sample was collected as a continuous variable by the trained nurses performing the blood sample collection in both HIV clinics.

### Outcomes

The primary outcomes of the study were feasibility, defined as the number of PSCs with at least one spot filled and that were able to be utilized for analysis, the overall acceptability of capillary whole blood sample collection with the PSC, and how many patients were provided with their results.

### Statistical Analyses

Standard descriptive statistics were used to report results generally. Mean, standard deviation and Student’s t-test were used for quantitative variables and frequency and chi-squared test were used for qualitative variables where the level of significance was set at < 0.05. A logistic regression model was run to explore the factors contributing to PSC acceptability. To assess the relationship between PSC acceptability and factors of interest (reported pain, reported easiness of the procedure, and reported procedure time), we used logistic regression models to generate adjusted odds ratios (aORs). The model for acceptance was adjusted for gender, age, and education. The level of significance was set at < 0.05, and data were analysed using Stata, version 16.

### Data Visualization

Box and whisker plots with the interquartile range were created to represent the time (minutes) for PSC sample collection.

### Ethical Considerations

This study received ethical clearance from the Ethical Committee of the Hospital Clínic de Barcelona, Barcelona, Spain (n. HCB/2020/1119), from Makerere University School of Medicine Research and Ethics Committee, Kampala, Uganda (n. Mak-SOMREC-2021-64), and the Joint Institutional Review Board for Animal & Human Bioethics (JIRB) of the University of Yaoundé I (UYI), Yaoundé, Cameroon (n. BTC-JIRB2022-030). This study was performed in accordance with relevant guidelines and regulations. All participants provided informed written consent. Study information sheets and informed consent forms were available in English, French, and Luganda.

### Patient and Public Involvement

Patients or the public were not involved in the design, or conduct, or reporting, or dissemination plans of our research at this time.

## Results

A total of 192 participants were included (88 in Uganda and 104 in Cameroon). The majority of them were female (113, 60.1%) with a mean age of 44.8 years (± SD ± 11.3), and had completed up to secondary school (101, 52.9%) **(**Table 1**).** All participants reported an HIV diagnosis, with the median year of diagnosis being 2013 (IQR = 2010–2018), and all were receiving antiretroviral therapy (ART) at the time of study participation. The overall HBsAg prevalence was 11.1% (95% CI 6.9–17.5) and 1.0% for HCV. Similar HBV prevalences were observed in Cameroon and Uganda, respectively (10.7% vs. 11.4%), and all participants had undetectable HBV-DNA levels. Cameroon reported two (3.5%) anti-HCV positive cases, of which, both were HCV-RNA negative. All participants were informed of their status and those who were HBsAg positive were referred to liver care.

**Table 1:** Key sociodemographic and potential risk factors of study participants, *n* = 192

	Overall (*N* = 192)		Cameroon (*N* = 104)		Uganda (*N* = 88)	
**Sex**	*N*	*%*	*N*	*%*	*N*	*%*
Male	75	39.9	33	31.7	42	47.7
Female	113	60.1	67	67	46	52.3
**Age**						
Mean	44.83 (± 11.26)		48.38 (± 10.90)		40.63 (± 10.23)	
18–92	13	6.8	4	3.9	9	10.2
30–39	55	28.7	19	18.3	36	40.9
40–49	56	29.2	32	30.8	24	27.3
50–59	43	22.4	31	29.8	12	13.6
≥ 60	25	13	18	17.3	7	8
**Ethnic group**						
Bamileke	24	12.6	24	23.3	-	-
Ewondo	17	2.1	17	16.5	-	-
Eton	4	2.1	4	3.9	-	-
Etong	4	2.1	4	3.9	-	-
Beti	4	2.1	4	3.9	-	-
Ganda	48	25.1	-	-	48	54.6
Nyoro	11	5.8	-	-	11	12.5
Soga	5	2.6	-	-	5	5.7
Nyakale	5	2.6	-	-	5	5.7
Toro	6	3.1	-	-	6	6.8
Other	68	35.6	50	48.5	18	20.5
**No. children**						
Mean	3.47 (± 2.40)		3.49 (± 2.34)		3.45 (± 2.46)	
0–2	57	38.8	22	36.1	35	40.7
3–5	67	45.6	26	42.6	41	47.7
6–10	20	13.6	12	19.7	8	9.3
> 10	3	2	1	1.6	2	2.3
**No. people living in the same household**						
Mean	4.38 (± 2.55)		5.15 (± 2.70)		3.62 (± 2.16)	
1–3 people	78	44.6	28	32.2	50	56.8
4–6 people	63	36	33	37.9	30	34.1
7–10 people	28	16	21	24.1	7	8
> 10 people	6	3.4	5	5.8	1	1.1
**Education**						
No education	17	8.9	13	12.6	4	4.6
Primary education completed	73	38.2	39	37.9	34	38.6
Secondary education completed	82	42.9	39	37.9	43	48.9
University (bachelor) completed	14	7.3	8	7.77	6	6.8
Vocational training completed	1	0.5	0	9	1	1.1
University (master or higher) completed	4	2.1	4	3.9	0	0
**Previous incarceration**						
Yes	27	14.2	0	0	27	31.4
No	163	85.8	104	100	59	68.6
**Traditional tattoo or scaring**						
Yes	12	6.2	7	6.74	5	5.7
No	176	93.6	93	89.4	83	94.3
**Prior surgery**						
Yes	9	4.7	2	1.9	7	7.9
No	181	95.3	100	98	81	92.1
**Hospitalization in past 2 years**						
Yes	15	7.9	6	5.8	9	10.3
No	176	92.2	98	94.2	78	89.7
**Herbal medicine use**						
Yes	54	28.4	19	18.6	35	39.8
No	136	71.6	83	81.4	53	60.2
**Hepatitis B vaccination**						
Yes	13	6.8	7	6.7	8	9.1
No	163	84.9	81	77.9	80	90.9
Not sure	16	8.3	16	15.4	0	0
**Previously tested for HBV**						
Yes	54	28.4	8	9.1	8	9.1
No	128	67.4	80	90.9	80	90.9
Not sure	8	4.2	0	0	0	0
**Previously tested for HCV**						
Yes	54	28.7	54	53.5	0	0
No	127	67.6	40	39.6	87	100
Not sure	7	3.7	7	6.9	0	0

*Missing values not reported here

Overall, most participants had heard of HBV or HCV before (153, 80.5%). Knowledge of HBV in Cameroon was higher than in Uganda (87.3% vs. 72.7%, *p* = 0.012), while knowledge of HCV was lower overall, and particularly in Cameroon (52.2% in Cameroon vs. 63.6% in Uganda). Participants in Cameroon more frequently reported having been tested for HBV and HCV before, in comparison to those in Uganda (45.1% vs. 9.1% for HBV and 53.5% vs. 0% for HCV, respectively).

### Acceptability

**Fig. 1:**
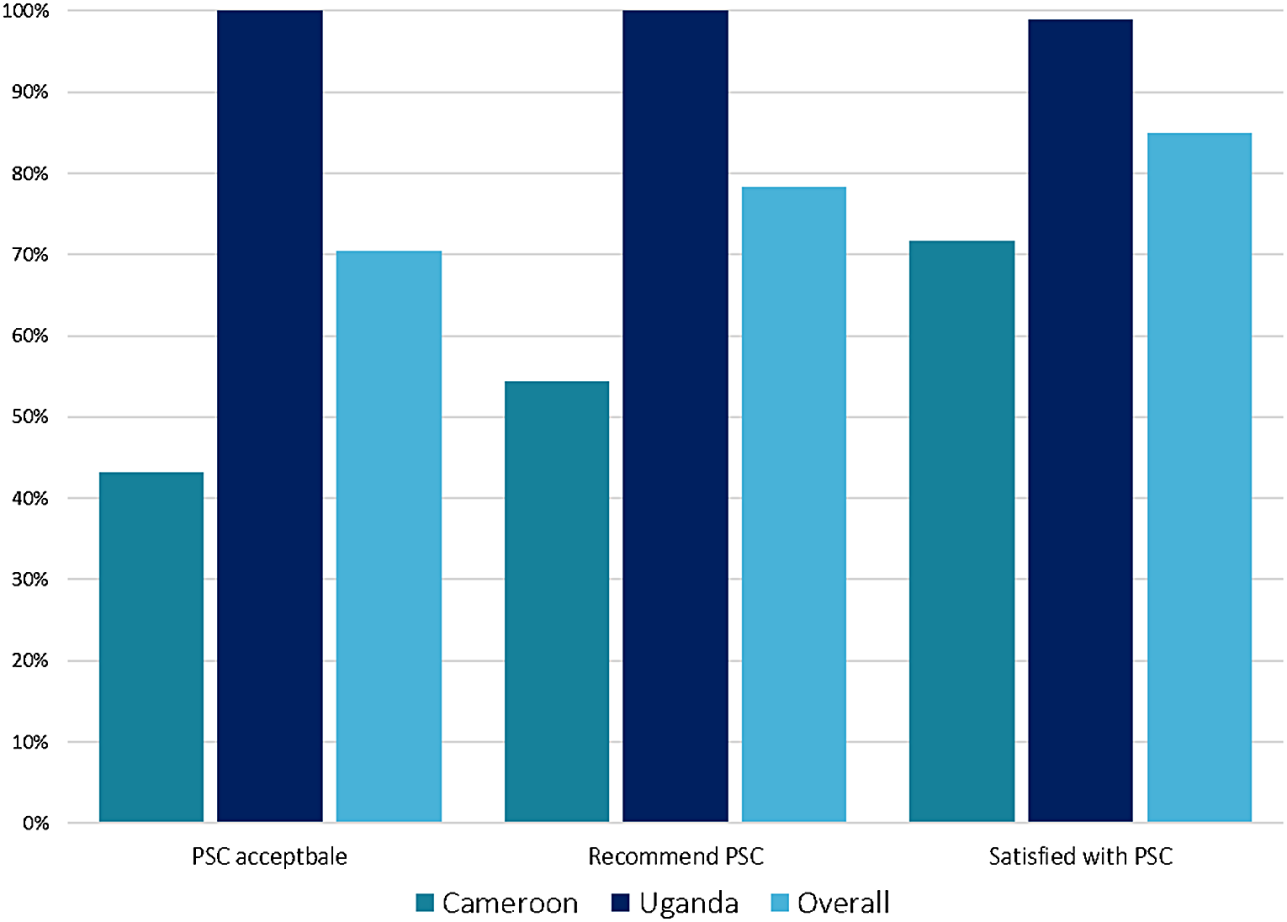
Proportion of participants reporting acceptability, recommendation, and satisfaction with the plasma separation card (PSC), overall and by country (*n* = 192)

Overall, nearly three quarters of respondents (129, 70%) reported that the PSC was an acceptable tool for viral hepatitis screening. The PSC was significantly more accepted in Uganda compared to Cameroon (100% vs. 43.2%, *p* < 0.001) and similarly, participants were more likely to recommend the PSC for viral hepatitis screening in Uganda than Cameroon (100% vs. 54.4%, *p* < 0.001). Over four-fifths of the participants (153, 85%) reported being satisfied with the PSC sample collection method. Nine out of ten Ugandan participants (78, 89.8%) reported the highest level of satisfaction with the PSC in comparison to 8.7% (*N* = 8) in Cameroon (Fig. 1). When asked if, in the future, they are given the option to receive testing by PSC, venepuncture or RDT, the majority (91, 50.3%) reported preferring PSC collected by capillary blood followed by RDT (42.5%). Differences in the choice of testing were observed between countries, with 64.8% of Ugandan respondents preferring the PSC for future testing in comparison to 36.6% of participants in Cameroon.

### Sample Collection Ease

**Fig. 2:**
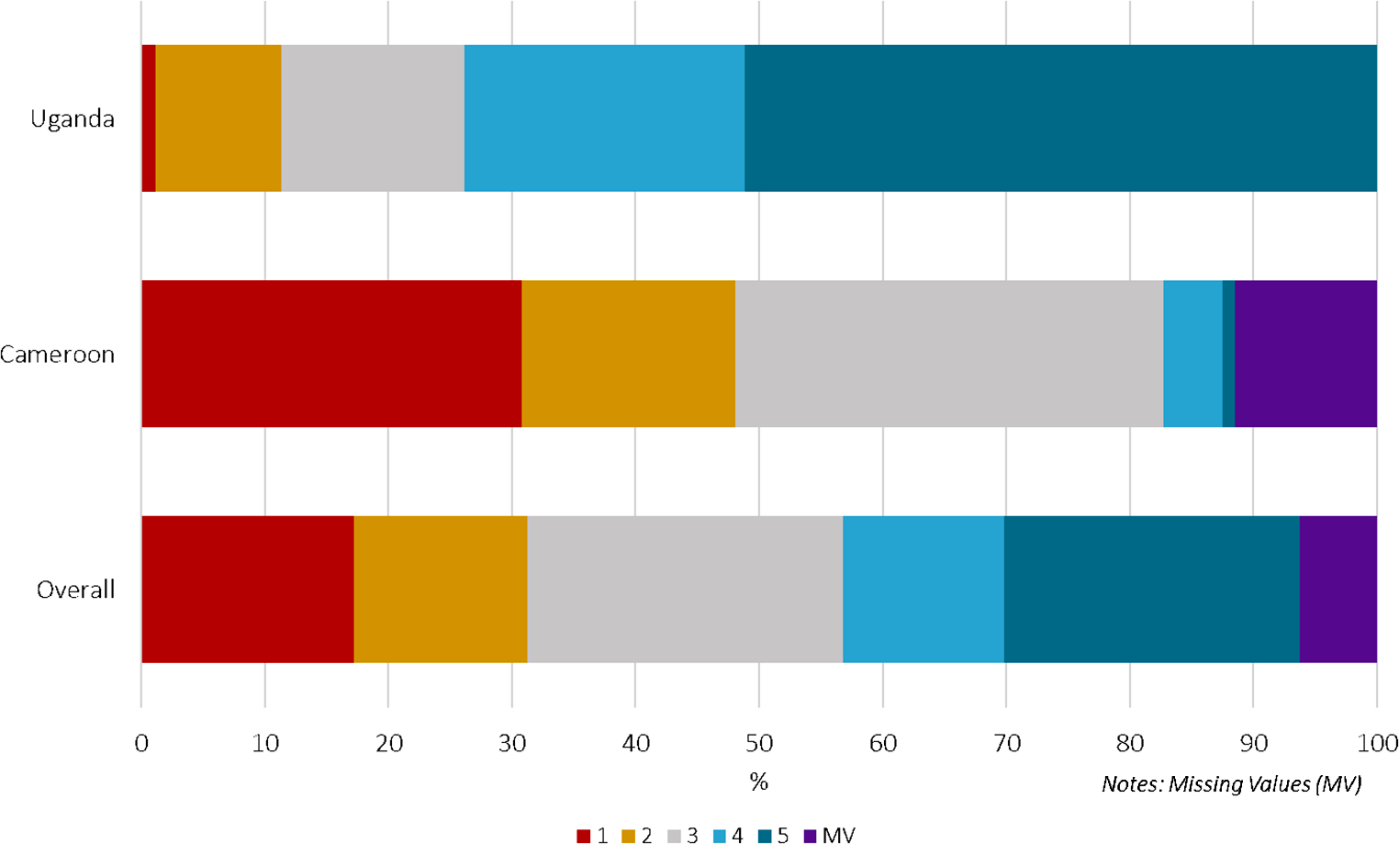
Level of self-reported ease utilizing and PSC from “not at all easy” (1) to “very easy” (5), overall and by country (*n* = 192)

Similar trends were observed when participants evaluated the ease of utilizing the PSC; 39.5% (*N* = 71) considered the PSC to be an “easy” or “very easy” collection method, of which 65 participants were Ugandan and six were Cameroonian, corresponding to 73.9% and 6.5% of their respective populations (Fig. 2).

**Fig. 3:**
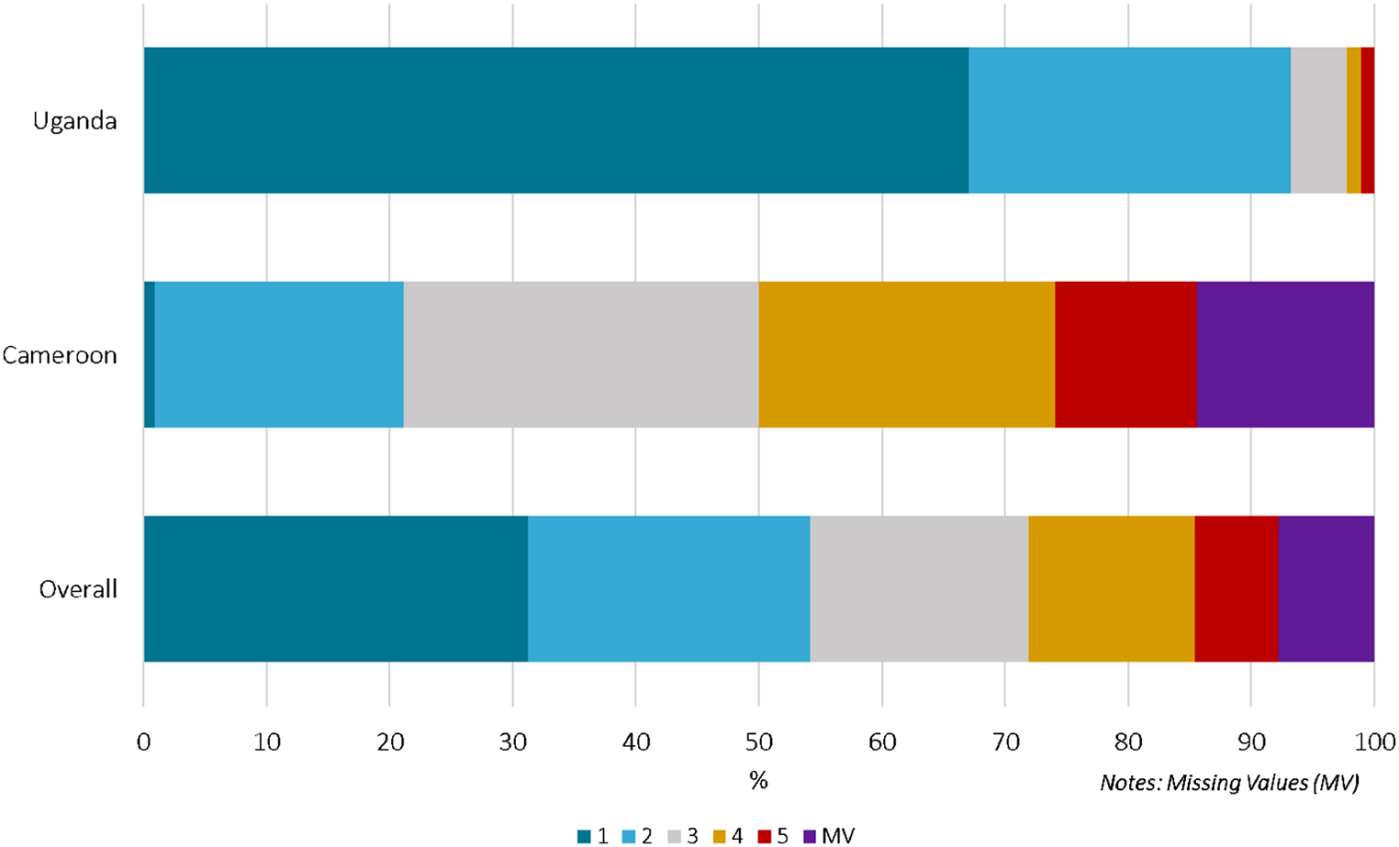
Level of self-reported pain utilizing the PSC from “not at all” (1) to “very painful” (5), overall and by country (*n* = 192)

In terms of pain, most participants (58.8%) reported “not at all” (60, 33.9%) or “somewhat painful” (44, 24.9%). Further, a total of thirteen participants (7.34%) described the method as “very painful”, corresponding to one participant in Uganda (1.1%) and 12 participants in Cameroon (14.5%) (Fig. 3).

### Feasibility

**Fig. 4:**
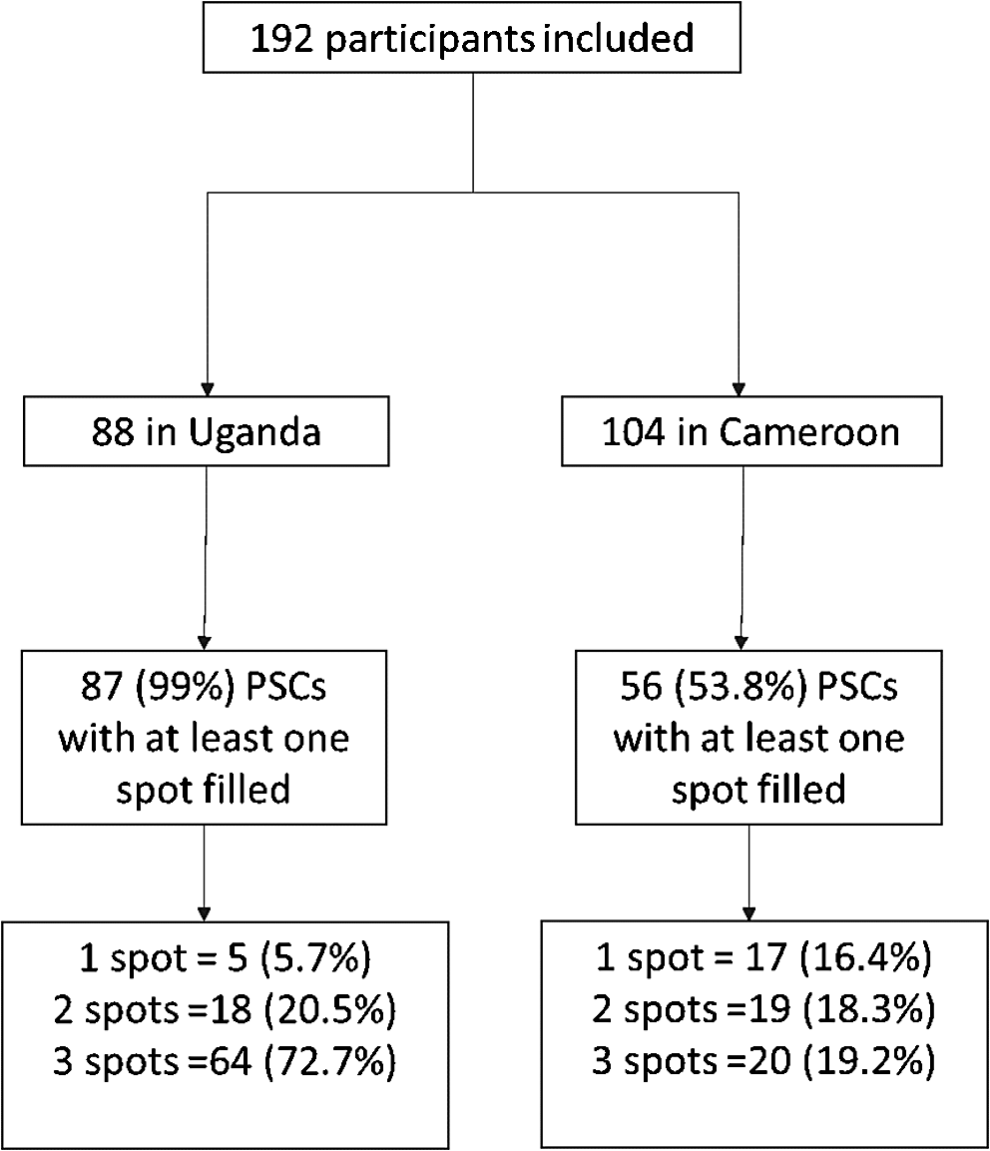
Plasma separation card sample viability, by country

Overall, 74.5% (*N* = 143) of PSCs had at least one spot sample filled and were viable for analysis. Only one sample of the total 88 samples collected in Uganda did not have any of the three blood spots correctly filled, thus 99% (*N* = 87) of the samples collected on the PSCs included at least one filled blood spot able to be utilized for analysis, whereas 53.4% (*N* = 56) samples collected in Cameroon included at least one filled blood spot. Further, among the PSCs collected (*N* = 143), 84 (58.9%) of them were adequately collected by healthcare workers (HCWs) and had all three viable spots completed. In Uganda, attaining three viable spots was seen in 73% (*N* = 64) of samples in comparison to 19.2% (*N* = 20) in Cameroon (Fig. 4).

**Fig. 5:**
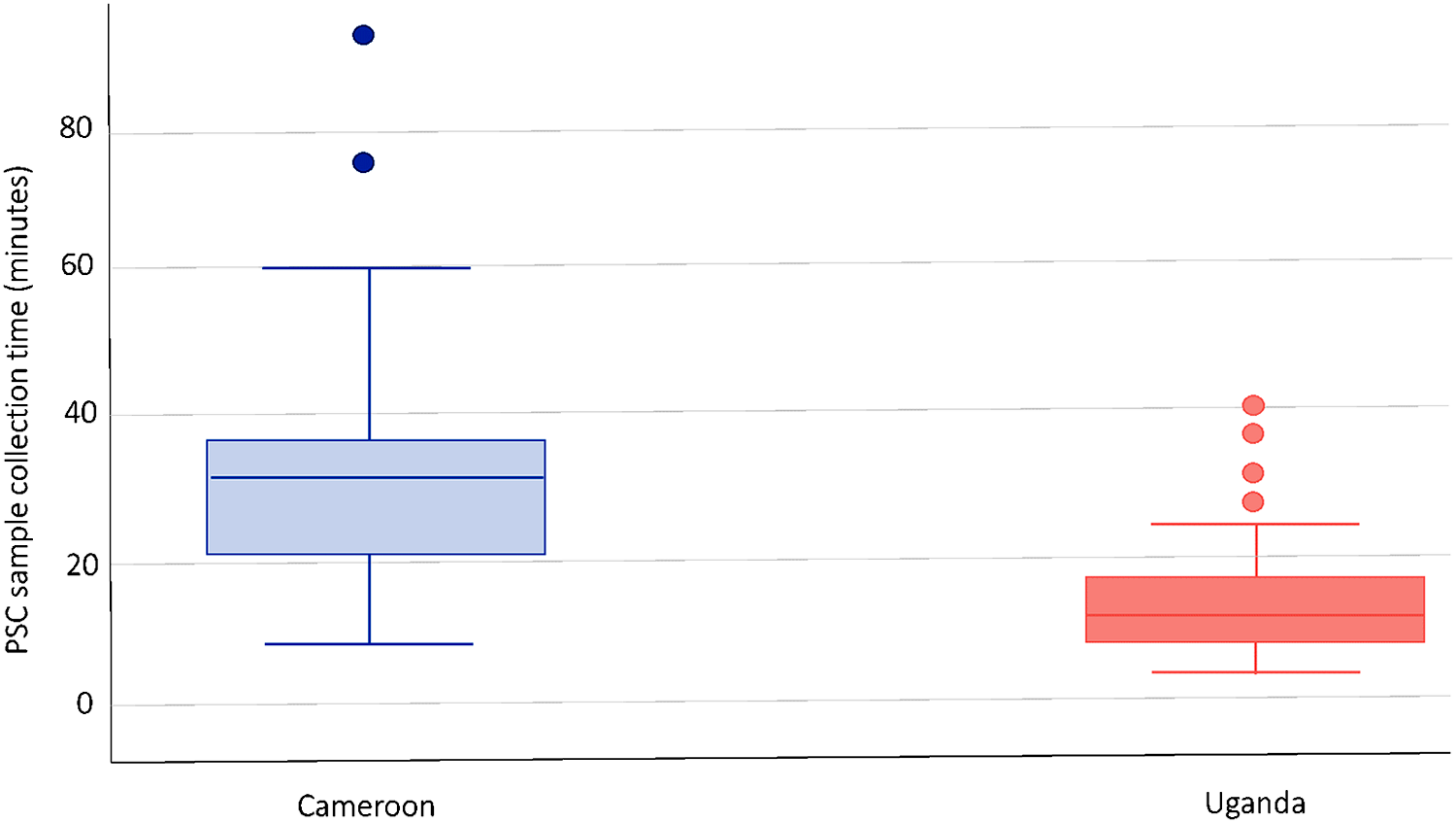
Box and whisker chart of average time to complete the PSC sample collection by country, *n* = 143

The mean sample collection duration was 21 min (IQR: 8–30), however the mean duration of time it took to collect samples was 30.5 ± 1.4 min in Cameroon vs. 9.8 ± 1.1 min in Uganda (*p* < 0.001) (Fig. 5). 33.5% (*N* = 61) of participants reported that blood sample collection should take less than 30 min (between 20 and 30 min), and 66.5% (*N* = 121) reported that the PSC should take less than 20 min.

### Logistic Regression

After adjustments in the logistic regression exploring factors contributing to PSC acceptability, reported easiness of method performance (aOR: 24.77 95% CI 2.97-206.42, *p* = 0.003) and reduced sample collection time (aOR: 3.73 95% CI 1.26–11.04, *p* = 0.017) (Table 2) were associated with greater odds of PSC acceptance. Reported pain did not significantly decrease odds of PSC acceptability (*p* = 0.686).

**Table 2:** Crude and adjusted odds ratios evaluating plasma separation card (PSC) accepted among study participants in cameroon and Uganda

Acceptability	Crude Odds ratio (cOD)	P-value	95% Conf. Interval	Adjusted Odds ratio (aOR)*	p-value	95% Conf. Interval
*Reported pain*	1.13	0.792	0.46–2.75	1.21	0.678	0.49–3.01
*Reported easiness*	13.28	0.001	2.74–64.3	24.77	0.003	2.97-206.42
*Quicker extraction time*	4.02	0.008	1.43–11.29	3.73	0.017	1.26–11.04

*Adjusted for gender, age, and education level. Significance set at *p* < 0.01

## Discussion

This was the first study evaluating the feasibility and acceptability of using the plasma separation card (PSC) for viral hepatitis screening among PLVIH in Cameroon and Uganda. Overall, 74.5% of the samples had at least one spot filled and were viable for analysis and acceptability by users of the tool and was higher in Uganda (100%) in comparison to Cameroon (43.2%), suggesting that further research is needed in Cameroon to understand why this was the case.

Studies evaluating the use of standard DBS are well described in the literature for viral hepatitis and HIV, particularly in LMICs. To our knowledge, no real-world studies evaluating the PSC for HBV have been carried out in sSA. One study carried out by the Foundation for Innovative New Diagnostics (FIND) evaluating samples obtained with the PSC for HCV RNA quantification had clinical sites in four countries: Cameroon, Georgia, Greece, and Rwanda but were all analyzed in a central laboratory in Australia with samples obtained through standard phlebotomy and fingerstick to compare [23]. Our study found that two-thirds of the samples collected with the PSC fulfilled quality standards and were viable for analysis with at least one out of three spots filled, although this was substantially less in Cameroon than in Uganda.

These results reporting feasibility differ from studies evaluating standard DBS collection, particularly in other African settings. For example, one study in Zambia reported that 98% of DBS samples collected by non-medical personnel for HIV viral load monitoring were done so correctly [24]. The differences observed between the two countries in our study warrant further analysis given that each country is made up of a heterogeneous population with varying HCW personnel, infrastructure, climate, and ethnic groups. Differences observed in our study point out the necessity to evaluate and assess potential external factors contributing to the feasibility of DBS testing with the PSC in resource-limited settings, despite very successful reported results in certain settings, such as Uganda and Zambia, with regular DBS.

Regarding the acceptability of utilizing PSCs, given that no other study reporting it currently exists in the published literature, results cannot be compared to other similar studies. Acceptability of the PSC overall was 70%, reaching 100% in the Ugandan setting. This is congruent with the existing literature on acceptability of DBS testing in African settings, where good acceptability of capillary blood sampling from DBS in comparison to venepuncture is reported [25]. When evaluating acceptability of point-of-care (PoC) testing for HBV testing in other LMICs, acceptability of these strategies, including rapid testing by fingerstick, was around 70% as well [20]. This acceptability coverage is similar to that reported in other sSA countries for diseases like HIV (63-86%) [26, 27] or malaria (64%) [28].

In Cameroon, however, acceptability was substantially lower, which could, in part, be a result of the increased average sample collection time observed. Consistently, after adjustments, reduced sample collection time was associated with greater odds of acceptability overall. Despite receiving the same training and informational material in each setting, additional external factors might have influenced the performance of HCW in each setting, leading to an increased extraction time in Cameroon. These factors could include individual HCW factors such as years of experience or previous experience in capillary blood sample collection, along with the level of acquired knowledge resulting from the training sessions or factors related to the burden of their workload. Further, additional factors include patient level factors such as challenging fingers (due to poor circulation or calluses) for capillary blood sample obtention that may have required sticking more than one finger.

Similarly, rural facilities are often unable to offer adequate “in-service” staff training, and can only afford training for a reduced number of staff members in large urban centers, who later provide cascade training to the rest of the team. As reported by Smit et al. [29], that “in-service” cascading of skill is oftentimes of poorer quality and thus not as effective. While both settings included in this study were found in urban centers, this is important to consider for possibly scaling up the tool given that this lack of effective and standardized training, knowledge, and skills on DBS sample collection is likely to be essential in the effective use of this method [29].

Despite the increased time in sample collection seen in Cameroon, sample collection time was aligned with the overall preferences of one third of participants, who responded that blood sample collection should take less than 30 min. In Cameroon particularly, 60% of participants reported blood sample collection should take less than 30 min. Therefore, lower rates of acceptability observed in Cameroon may be due to an external factor not directly measured within this study.

Further, reported easiness of the method by participants was also found to be associated with greater odds of acceptance. However, reported pain was not observed to have a significant association. These results suggest that the experienced pain may not have been a limiting factor to considering the PSC as an acceptable blood collection method for viral hepatitis testing. The participants’ attitudes towards the procedure, such as fear, potentially conditioned by their previous experiences on blood extraction procedures, may have been contributing factors to reporting the grade of easiness of the PSC method.

Increasing knowledge on viral hepatitis and its consequences may contribute to increased acceptability. Knowledge of HBV in Cameroon was high overall, and higher than knowledge of HCV. However, despite being aware of the infections, overall, testing for both HBV and HCV was low. This drop-off from knowledge to testing uptake may be due to the fact that testing for viral hepatitis is paid for out of pocket by patients, making it inaccessible to most, despite being engaged in regular HIV care. Despite engagement with the health system for treatment of their HIV, referrals to liver specialists were provided those who were HBsAg positive in order to ensure proper monitoring of the liver. The median year of diagnosis and treatment initiation was 2013, suggesting that antiviral treatment may have suppressed HBV-DNA levels (given that none had detectable levels on PSC); however, elimination of the risk of liver disease progression among those who are co-infected is not eliminated [30, 31] and therefore, ensuring linkage to liver specialist care is essential.

Notably, there was a pooled prevalence of 11.1% of HBsAg positivity among those screened and 1% anti-HCV positivity, of which both cases were found in Cameroon. These data are in line with prevalence data from both countries, which further reinforce the need for increased viral hepatitis testing in these countries. However, it is important to note that use of the PSC for viral hepatitis testing was used off-label as the PSC currently only has approval for HIV viral load quantification.

Our study has several strengths, including the standardisation of the PSC across two different settings and the novelty of being the first study evaluating the feasibility and acceptability of its use through fingerstick collection among users. The PSC is particularly appealing for blood sample collection in resource-limited settings given the ability to separate plasma from whole blood without the need for centrifugation or cold chain storage. However, the study has several limitations which need to be taken into account when interpreting the results. Firstly, platforms to analyse dried plasma samples from PSCs in Cameroon and Uganda were limited in-country. Despite Uganda being able to process both serological and molecular samples in-country, molecular testing in Cameroon needed to be carried out outside of the country (in Barcelona, Spain), limiting the generalizability of results for real-world settings. Similarly, sensitivity and specificity of analysing samples from PSCs have been documented, and are high [22], but not 100%, making false positives and negatives a concern. Specifically, HBsAg and anti-HCV sensitivity on PSC has been reported to be 98%, with specificity increasing to 100% in the case of anti-HCV testing and slightly decreasing to 96% for HBsAg [22]. In our study, results were not compared to another method (e.g., standard phlebotomy) and test results were taken at face value. In those lines, the analysis of Cameroon samples performed in Barcelona revealed a differing HBsAg result in one of the six samples, which may be interpreted as a false positive. This highlights the importance of understanding the sensitivity and specificity of these analyses in further studies and when implementing testing strategies with PoC tests or DBS. Furthermore, the participants were PLHIV who, on average, have been on ART since 2013 and, therefore, the undetectable HBV-DNA documented among those HBsAg positive in our study could be due to viral suppression from long-term ART. Importantly, this was a cross-sectional study in two different settings and, therefore, the results cannot be generalised to other parts of Cameroon or Uganda or to other African countries.

## Conclusion

Dried plasma samples collected from capillary blood collected on the plasma separation card is an alternative method for viral hepatitis testing which this study found to be acceptable and feasible in Uganda as compared to Cameroon. Further assessment of potentially determining factors, such as service delivery infrastructure, HCW training and experience, and population predisposition to the method should be considered.

## Data Availability

The data that support the findings of this study are not openly available but are available from the corresponding author upon request.

## Abbreviations

Anti-HCV: anti-HCV antibodies
ART: Antiretroviral therapy
CHUY: University Teaching Hospital of Yaoundé
CPHL: Central Public Health Laboratory
CE: Conformité Européenne
DBS: Dried blood spot
FIND: Foundation for Innovative New Diagnostics
HBsAg: Hepatitis B surface antigen
HBV: Hepatitis B virus
HCV: Hepatitis C virus
HCW: Healthcare worker
JIRB: Joint Institutional Review Board for Animal & Human Bioethics
PLVIH: People living with HIV
PoC: Point-of-care
PSC: Plasma separation card
sSA: sub-Saharan Africa
WHO: World Health Organization

## References

[1] Polaris Observatory Collaborators. Global prevalence, treatment, and prevention of hepatitis B virus infection in 2016: a modelling study. Lancet Gastroenterol Hepatol. 2018. 10.1016/S2468-1253(18)30056-6. 10.1016/S2468-1253(18)30056-6 29599078

[2] World Health Organization. Global progress report on HIV, viral hepatitis and sexually transmitted infections, 2021: accountability for the global health sector strategies 2016–2021: actions for impact. In: World Health Organization. 2021. https://apps.who.int/iris/bitstream/handle/10665/341412/9789240027077-eng.pdf. Accessed 18 May 2023.

[3] Cooke GS, Andrieux-Meyer I, Applegate TL, Atun R, Burry JR, Cheinquer H, et al. Accelerating the elimination of viral hepatitis: a Lancet Gastroenterology & Hepatology Commission. Lancet Gastroenterol Hepatol. 2019. 10.1016/S2468-1253(18)30270-X. 31669194 10.1016/S2468-1253(19)30319-X

[4] Bigna JJ, Amougou MA, Asangbeh SL, Kenne AM, Nansseu JR. Seroprevalence of hepatitis C virus infection in Cameroon: a systematic review and meta-analysis. BMJ Open. 2017. 10.1136/bmjopen-2016-015748. 28851778 10.1136/bmjopen-2016-015748 PMC5724202

[5] Njouom R, Siffert I, Texier G, Lachenal G, Tejiokem MC, Pépin J, et al. The burden of hepatitis C virus in Cameroon: spatial epidemiology and historical perspective. J Viral Hepat. 2018. 10.1111/jvh.12894. 29533500 10.1111/jvh.12894

[6] Kowo MP, Andoulo FA, Ngek LT, Sizimboue DT, Ndam AN, Ondo BE, et al. Prevalence of hepatitis C virus and associated risk factors among inmates at new bell prison, Douala, Cameroon. Open J Epidemiol. 2019. 10.4236/ojepi.2019.92011.

[7] Kafeero HM, Ndagire D, Ocama P, Kudamba A, Walusansa A, Sendagire H. Prevalence and predictors of hepatitis B virus (HBV) infection in east Africa: evidence from a systematic review and meta-analysis of epidemiological studies published from 2005 to 2020. Arch Public Health. 2021. 10.1186/s13690-021-00686-1. 34537079 10.1186/s13690-021-00686-1 PMC8449462

[8] Mastroianni CM, Lichtner M, Mascia C, Zuccalà P, Vullo V. Molecular mechanisms of liver fibrosis in HIV/HCV coinfection. Int J Mol Sci. 2014. 10.3390/ijms15069184. 24865485 10.3390/ijms15069184 PMC4100089

[9] Parvez MK. HBV and HIV co-infection: impact on liver pathobiology and therapeutic approaches. World J Hepatol. 2015. 10.4254/wjh.v7.i1.121. 25625003 10.4254/wjh.v7.i1.121 PMC4295189

[10] World Health Organization. Global Hepatitis Programme. Global Health Sector Strategy on Viral Hepatitis, 2016–2021: Towards Ending Viral Hepatitis. In: World Health Organization. 2016. https://www.who.int/publications/i/item/WHO-HIV-2016.06.

[11] Chevaliez S, Pawlotsky JM. New Virological tools for screening, diagnosis and monitoring of hepatitis B and C in resource-limited settings. J Hepatol. 2018;69(4):916–26. 29800630 10.1016/j.jhep.2018.05.017

[12] Nichols BE, Girdwood SJ, Shibemba A, Sikota S, Gill CJ, Mwananyanda L, et al. Cost and impact of dried blood spot Versus plasma separation card for scale-up of viral load testing in resource-limited settings. Clin Infect Dis. 2020. 10.1016/j.jhep.2018.05.017. 31321438 10.1093/cid/ciz338 PMC7931834

[13] World Health Organization. Guidelines on Hepatitis B and C Testing. In: World Health Organization. 2017. https://www.who.int/publications/i/item/9789241549981.

[14] Kenmoe S, Tagnouokam PAN, Nde CK, Mella-Tamko GF, Njouom R. Using dried blood spot for the detection of HBsAg and anti-HCV antibodies in Cameroon. BMC Res Notes. 2018. 10.1186/s13104-018-3931-3. 30446000 10.1186/s13104-018-3931-3 PMC6240176

[15] Parr JB, Lodge EK, Holzmayer V, Pepin J, Frost EH, Fried MW, et al. An efficient, Large-Scale Survey of Hepatitis C Viremia in the Democratic Republic of the Congo using dried blood spots. Clin Infect Dis. 2018. 10.1093/cid/cix771. 29048459 10.1093/cid/cix771 PMC5850542

[16] Lange B, Roberts T, Cohn J, Greenman J, Camp J, Ishizaki A, et al. Diagnostic accuracy of detection and quantification of HBV-DNA and HCV-RNA using dried blood spot (DBS) samples - a systematic review and meta-analysis. BMC Infect Dis. 2017. 10.1186/s12879-017-2776-z. 29143616 10.1186/s12879-017-2776-z PMC5688458

[17] Ishizaki A, Bouscaillou J, Luhmann N, Liu S, Chua R, Walsh N, et al. Survey of programmatic experiences and challenges in delivery of hepatitis B and C testing in low- and middle-income countries. BMC Infect Dis. 2017. 10.1186/s12879-017-2767-0. 29143609 10.1186/s12879-017-2767-0 PMC5688462

[18] Carmona S, Seiverth B, Magubane D, Hans L, Hoppler M. Separation of plasma from whole blood by Use of the cobas plasma separation card: a Compelling Alternative to dried blood spots for quantification of HIV-1 viral load. J Clin Microbiol. 2019. 10.1128/JCM.01336-18. 30728197 10.1128/JCM.01336-18 PMC6440768

[19] Vubil A, Zicai AF, Sitoe N, Nhachigule C, Meggi B, Loquiha O, et al. Accurate HIV viral load measurement in primary health care settings using the cobas® plasma separation card. PLoS ONE. 2020. 10.1371/journal.pone.0232122. 32374748 10.1371/journal.pone.0232122 PMC7202605

[20] Lemoine M, Shimakawa Y, Njie R, Taal M, Ndow G, Chemin I, et al. Acceptability and feasibility of a screen-and-treat programme for hepatitis B virus infection in the Gambia: the Prevention of Liver Fibrosis and Cancer in Africa (PROLIFICA) study. Lancet Global Health. 2016. 10.1016/S2214-109X(16)30130-9. 27443781 10.1016/S2214-109X(16)30130-9

[21] Ministerio de Sanidad. Boletín Oficial del Estado (BOE) núm. 176. BOE-A-2021-12405. In: Agencia Estatal Boletín oficial del Estado. 2021. https://www.boe.es/buscar/act.php?id=BOE-A-2021-12405.

[22] Martínez-Campreciós J, Rando-Segura A, Buti M, Rodrigo-Velásquez F, Riveiro-Barciela M, Barreira-Díaz A, et al. Reflex viral load testing in dried blood spots generated by plasma separation card allows the screening and diagnosis of chronic viral hepatitis. J Virol Methods. 2021. 10.1016/j.jviromet.2020.114039. 33338545 10.1016/j.jviromet.2020.114039

[23] Malobela A, Amougou M, Ilioupoulos P, Mugisha JC, Berishvili N, Sologashvili M et al. Diagnostic accuracy of dried blood spot and plasma separation cards samples for testing hepatitis C virus RNA. In: International Liver Congress 2021. 2021. https://www.postersessiononline.eu/173580348_eu/congresos/ILC2021/aula/-PO_908_ILC2021.pdf.

[24] Sikombe K, Hantuba C, Musukuma K, Sharma A, Padian N, Holmes C, et al. Accurate dried blood spots collection in the community using non-medically trained personnel could support scaling up routine viral load testing in resource limited settings. PLoS ONE. 2019. 10.1371/journal.pone.0223573. 31622394 10.1371/journal.pone.0223573 PMC6797100

[25] Tuaillon E, Kania D, Pisoni A, Bollore K, Taieb F, Ontsira Ngoyi EN, et al. Dried blood spot tests for the diagnosis and therapeutic monitoring of HIV and viral Hepatitis B and C. Front Microbiol. 2020. 10.3389/fmicb.2020.00373. 32210946 10.3389/fmicb.2020.00373 PMC7075356

[26] Chamie G, Kwarisiima D, Clark TD, Kabami J, Jain V, Geng E, et al. Uptake of community-based HIV testing during a multi-disease health campaign in rural Uganda. PLoS ONE. 2014. 10.1371/journal.pone.0084317. 24392124 10.1371/journal.pone.0084317 PMC3879307

[27] González R, Munguambe K, Aponte J, Bavo C, Nhalungo D, Macete E, et al. High HIV prevalence in a southern semi-rural area of Mozambique: a community-based survey. HIV Med. 2012. 10.1111/j.1468-1293.2012.01018.x. 22500780 10.1111/j.1468-1293.2012.01018.x

[28] Cook J, Xu W, Msellem M, Vonk M, Bergström B, Gosling R, et al. Mass screening and treatment on the basis of results of a Plasmodium falciparum-specific rapid diagnostic test did not reduce malaria incidence in Zanzibar. J Infect Dis. 2015. 10.1093/infdis/jiu655. 25429102 10.1093/infdis/jiu655 PMC10881232

[29] Smit PW, Sollis KA, Fiscus S, Ford N, Vitoria M, Essajee S, et al. Systematic review of the use of dried blood spots for monitoring HIV viral load and for early infant diagnosis. PLoS ONE. 2014. 10.1371/journal.pone.0086461. 24603442 10.1371/journal.pone.0086461 PMC3945725

[30] Surial B, Ramírez Mena A, Roumet M, Limacher A, Smit C, Leleux O, et al. External validation of the PAGE-B score for HCC risk prediction in people living with HIV/HBV coinfection. J Hepatol. 2023. 10.1016/j.jhep.2022.12.029. 36690280 10.1016/j.jhep.2022.12.029

[31] Kim HN, Newcomb CW, Carbonari DM, Roy JA, Torgersen J, Althoff KN, et al. Risk of HCC with Hepatitis B Viremia among HIV/HBV-Coinfected persons in North America. Hepatology. 2021. 10.1002/hep.31839. 33780007 10.1002/hep.31839 PMC8843101

